# Artificial Intelligence to support ethical decision-making for incapacitated patients: a survey among German anesthesiologists and internists

**DOI:** 10.1186/s12910-024-01079-z

**Published:** 2024-07-18

**Authors:** Lasse Benzinger, Jelena Epping, Frank Ursin, Sabine Salloch

**Affiliations:** 1 Institute for Ethics, History and Philosophy of Medicine, Hannover Medical School (MHH), Carl-Neuberg-Str. 1, Hannover, 30625 Germany; 2 Department of Medical Sociology, Hannover Medical School (MHH), Carl-Neuberg-Str. 1, Hannover, 30625 Germany

**Keywords:** Artificial intelligence, Bioethics, Preference prediction, Incapacitated patients, Survey

## Abstract

**Background:**

Artificial intelligence (AI) has revolutionized various healthcare domains, where AI algorithms sometimes even outperform human specialists. However, the field of clinical ethics has remained largely untouched by AI advances. This study explores the attitudes of anesthesiologists and internists towards the use of AI-driven preference prediction tools to support ethical decision-making for incapacitated patients.

**Methods:**

A questionnaire was developed and pretested among medical students. The questionnaire was distributed to 200 German anesthesiologists and 200 German internists, thereby focusing on physicians who often encounter patients lacking decision-making capacity. The questionnaire covered attitudes toward AI-driven preference prediction, availability and utilization of Clinical Ethics Support Services (CESS), and experiences with ethically challenging situations. Descriptive statistics and bivariate analysis was performed. Qualitative responses were analyzed using content analysis in a mixed inductive-deductive approach.

**Results:**

Participants were predominantly male (69.3%), with ages ranging from 27 to 77. Most worked in nonacademic hospitals (82%). Physicians generally showed hesitance toward AI-driven preference prediction, citing concerns about the loss of individuality and humanity, lack of explicability in AI results, and doubts about AI’s ability to encompass the ethical deliberation process. In contrast, physicians had a more positive opinion of CESS. Availability of CESS varied, with 81.8% of participants reporting access. Among those without access, 91.8% expressed a desire for CESS.

Physicians' reluctance toward AI-driven preference prediction aligns with concerns about transparency, individuality, and human-machine interaction. While AI could enhance the accuracy of predictions and reduce surrogate burden, concerns about potential biases, de-humanisation, and lack of explicability persist.

**Conclusions:**

German physicians frequently encountering incapacitated patients exhibit hesitance toward AI-driven preference prediction but hold a higher esteem for CESS. Addressing concerns about individuality, explicability, and human-machine roles may facilitate the acceptance of AI in clinical ethics. Further research into patient and surrogate perspectives is needed to ensure AI aligns with patient preferences and values in complex medical decisions.

**Supplementary Information:**

The online version contains supplementary material available at 10.1186/s12910-024-01079-z.

## Introduction

Artificial intelligence (AI) executes tasks that formerly required human intelligence; consequently, new courses of action have emerged in healthcare. Algorithms perform in data-driven medical specialties, such as radiology or pathology [1], at the same or an even higher level than specialists do [2], especially in repetitive tasks that require little oversight and are time-consuming. Automated decision support is gaining importance not only in image-based medical specialties but also in other branches, such as surgery [3], pediatrics [4] or infection medicine [5]. AI-driven clinical decision support systems are, thus, increasingly developed and taken up in various diagnostic and therapeutic workflows.

Ethical decision-making in the clinic, by contrast, has so far been less affected by the progress in automated decision support. There seems to be a tendency to leave normatively laden decisions, for example, in intensive or end-of-life care, with human actors. This can be understood against the background of ethical decisions accompanied by high responsibility, the need for in-depth empathetic communication and the importance of contextual factors of each individual case. As a field known for its nonbinary way of decision-making, Clinical Ethics Support Services (CESS) have always profited from human intelligence and sympathy. According to Molewijk et al., CESS are “services that aim at supporting health-care professionals (including ancillary staff, managers, and directors), patients, and their families when being confronted with an ethical concern, question, or dilemma” [6]. The term is used for different kinds of institutionalized services that are operating within healthcare organizations, with ethical case deliberations being a main element [7]. Although ethics support structures have found their way into a plethora of clinics worldwide [8–10] where they have proven their worth for many stakeholders [11, 12], their availability varies throughout different countries [9, 13] and their utilization often appears to be lacking [14]. The unparalleled importance of individuality, autonomy and trust in as well as responsibility of counsellors in ethical support processes are aspects that people are reluctant to entrust to digital algorithms.

On the other hand, the idea of letting machines implement ethical frameworks is not new to the academic ethical community and formed a big discussion about “artificial moral agents” [15] or “moral machines” [16]. Researchers have proposed various ways to tackle ethical challenges in biomedicine and healthcare more recently by using AI [17, 18]. Some tools specifically directed at clinical ethical decision-making have been put forward: Rid and Wendler hypothesized a Patient Preference Predictor (PPP) to use when patients with temporarily or permanently impaired capacity are not able to verbalize their preferences concerning medical interventions. Based on, for example, healthcare records, ad hoc empirical studies and social media presences, the PPP supposedly predicts the sentiment towards (non-)treatment that an individual patient would have expressed (were they in the constitution to do so) more accurately than surrogates [19]. Lamanna and Byrne support this notion by suggesting the use of an “Autonomy Algorithm” quite similar to the PPP already mentioned [20]. Meier et al. recently developed the prototype of a medical ethical advisor called “METHAD” in a proof-of-concept study. The advisor operationalizes three out of the four principles of biomedical ethics according to Beauchamp and Childress (autonomy, beneficence, non-maleficence) [21] and gives general advice regarding different interventions based on case-specific user input [22]. Earp et al., finally, discuss options for complementing population-level data on patient preferences with documents that contain patient-specific data on individual reasons and values [23].

The use of AI for preference prediction in incapacitated patients as deeply personal and far-reaching choices might be motivated by several fundamental flaws in the conventional approach of surrogate decision-making through patient representatives: Empirical evidence indicates that surrogates frequently predict their loved one’s preferences inaccurately [24], and are vulnerable to internal or external bias [25, 26]. Furthermore, the decision-making itself has negative effects on stakeholders’ emotional and physical wellbeing [27, 28], and the emerging moral distress may cause healthcare professionals to consider changing their profession [29]. Advance directives that would facilitate the process are often inadequate, as they do not account for contextually changing preferences or were not formulated precisely and specifically enough to provide guidance in the concrete situation. In addition, they are often unavailable in the first place [30].

In this study, we explore the attitudes of physicians as the potential users of AI-driven preference prediction tools for supporting ethical decision-making. More specifically, internists and anesthesiologists were surveyed, as missing patient capacity is a more probable issue to them than to other specialties [31]. To the best of the authors’ knowledge, this is the first survey on physicians’ attitudes towards AI-driven tools for patient preference prediction. The questionnaire also included items covering the availability of CESS (specifically clinical ethics counselling) in the individual clinics, as well as attitudes towards the use of ethical support structures. The main aim of the study was to determine the willingness of the potential users to apply AI for ethical decision-making in their clinical routines, their hopes and reservations towards the new technology, and to compare these factors with related parameters concerning the use of CESS. Thereby, we hope to support the reasonable and worthwhile development of AI support tools for decision-making in incapacitated patients.

## Methods

The questionnaire was developed against the background of the research questions depicted above and pretested amongst medical students (*n* = 6). In the questionnaire, we provided a vignette on AI and patient preference prediction (see Table 1) to establish a common baseline understanding.

**Table 1:** Introductory vignette

*Imagine: In a few years, your hospital information system will contain a program to help you when making complicated ethical decisions (e.g. limiting therapy in intensive care). These are decisions for patients who cannot express their will themselves.*
*Based on clinical, demographic and personal characteristics from the medical record, the program predicts the preferences of incapacitated patients for an upcoming therapy decision. The prediction of the program is based on empirically collected training data processed by artificial intelligence.*
*The predictions generated by the program can be utilized to assist doctors or patient representatives in making decisions when there is no valid advance directive and the patients’ preferences are largely unknown.
*Artificial intelligence (AI) is the ability of a machine to imitate human competencies, such as logical thinking, learning, planning and creativity. It enables technical systems to find correlations in the data provided without explicit guidance and, based on these, to independently optimize their learning and work processes as well as their results.

Physicians were then asked to indicate their attitudes towards diverse ethical implications of the use of the PPP. A five-point Likert-scale was used to assess the attitudes, offering five options ranging from “absolutely disagree” (1) to “absolutely agree” (5), as well as the option to declare “no opinion.”

The questionnaire also included questions on the availability of CESS in the respondents’ respective clinic and, if available, whether they had already made use of it, or, if not, whether they wished to have access to clinical ethics support. Furthermore, an assessment of the physicians’ attitudes towards the use of CESS, the preference prediction tool and the subjects’ experiences with ethically challenging situations was conducted (see Supplement 1 “Questionnaire”).

Ultimately, the subjects were asked to write short free-text estimations regarding the chances and risks of the use of AI-enabled preference prediction in clinical decision-making. All questions were developed and presented in German.

The survey was distributed amongst the German DocCheck© physician panel until a predefined number of anesthesiologists and internists working in a clinical setting had completed the questionnaire. The composition of the sample aimed to represent healthcare professionals who most frequently encounter incapacitated patients and related uncertainties in therapy decisions. The DocCheck© physician panel consists of users of the DocCheck© website who are demonstrably members of the medical profession. The participants were recruited online. Doctors who expressed their interest in being contacted for survey research were e-mailed. They give their consent to participate in the present study and were compensated with a monetary amount of 10 EUR. Ethical approval for the survey was not required according to German law since the data collection took place anonymously [32, 33].

After completing the collection, the data was provided as a SPV file and analyzed using IBM SPSS Statistics 28. Various hypotheses were formulated to test differences in the response behavior of different groups of participants regarding ethically relevant topics. The t-test for two independent samples was used to compare the means of two different groups. Spearman’s rank correlation was used for the identification of association between two items. During the analysis, a *p*-value of < 0.05 was considered significant. The free text questions were analyzed referring to the method of qualitative text analysis according to Kuckartz [34] using MAXQDA 2018 Plus. The participants’ statements were screened for explicit reasons in favor of or against the use of AI in clinical ethical decision-making. The reasons identified in the mixed inductive-deductive approach were grouped into six predefined main categories (autonomy, beneficence, non-maleficence, justice, explicability, other), and then assigned finely granulated inductive codes that allowed for the detailed analysis of the participants’ hopes and fears.

## Results

### Sample characteristics

The resulting sample consisted of 200 anesthesiologists and 200 internists aged 27 to 77 (M = 45.01; SD = 10.226). Over two-thirds (69.3%) of respondents self-reported as male and 30.5% as female, one person did not identify as either of the binary genders. Compared to the German Medical Associations’ Physicians’ statistics of 2021 (Anesthesiology: 44.5% female, Inner Medicine: 41.0% female) [35], male participants were overrepresented in the sample. Participants were also generally younger than the German parent population (< 35 = 18.94%, 5–39 = 13.24%, 0–49 = 21.61%, 0–59 = 24.60%, 0–65 = 13.07%, 65 = 8.54%) [35] The majority of participants worked in nonacademic hospitals (82%), while the rest were employed in academic hospitals (17%) or rehabilitation clinics (1%) (see Table 2).

**Table 2:** Characterization of the sample

Measure	Item	*n*	Percent
Gender	Female	122	30.5
	Male	277	69.3
	Other	1	0.3
Age	< 35	59	14.8
	35–40	84	21.0
	40–49	130	32.5
	50–59	81	20.2
	60–65	38	9.5
	> 65	8	2
Discipline	Internal medicine	200	50
	Anesthesiology	200	50
Place of work	Academic hospital	68	17
	Nonacademic hospital	328	82
	Rehabilitation clinic	4	1

### Opinions on artificial intelligence

**Fig. 1:**
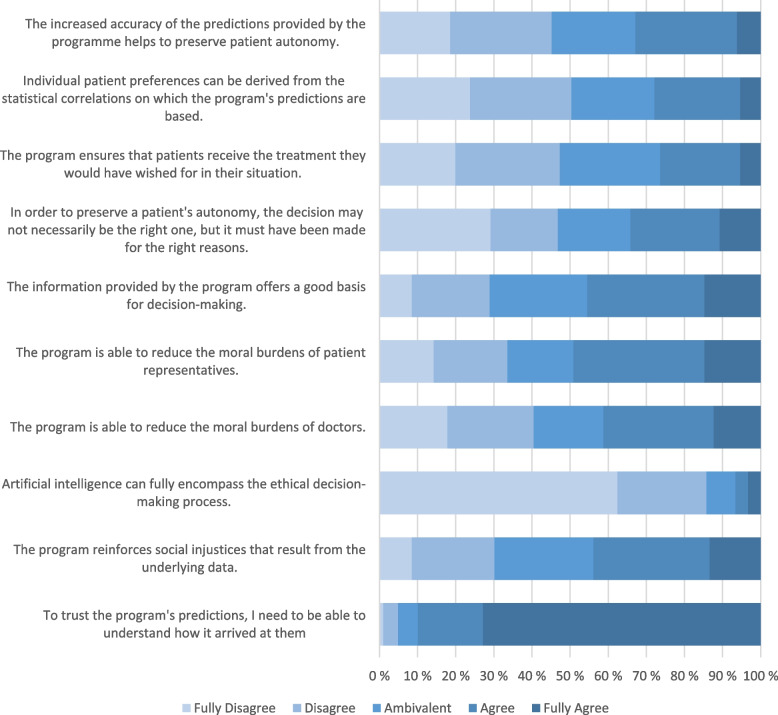
Responses to statements regarding AI-driven preference prediction

As a general result, the participants did not reject the use of AI-driven preference prediction to support clinical ethical decision-making, and, in some aspects, even welcomed it, but reserved a certain degree of scepticism. There was no significant difference in the acceptance of the program between genders, or anesthesiologists and internists. In addition, various topics, such as bias and potential shortcomings of computerized support, were approached with perceptible wariness. As a safeguard requisite, physicians agreed that the explicability of the novel tools is necessary. A depiction of the participants’ response behavior can be found in Fig. 1.

As ethically complex situations arise on a regular basis in clinical routine, we will shed more light on this topic by correlating items regarding the emotional strain induced by them to the physicians’ answers. Participants who reportedly found these situations to be stressful had a slightly more favorable view of patient preference prediction (ρ = 0.10; *p* = .028). In addition, the more a participant believed the prediction to be useful for doctors, the more they expected it to be useful for patient representatives (ρ = 0.68; *p* < .001). Individuals with a higher priority for explicability in computer predictions were less likely to accept the use of AI-driven preference prediction (ρ = − 0.14; *p* = .002), and more likely to endorse CESS (ρ = 0.24; *p* < .001).

### Opinions on clinical ethics support services

**Fig. 2:**
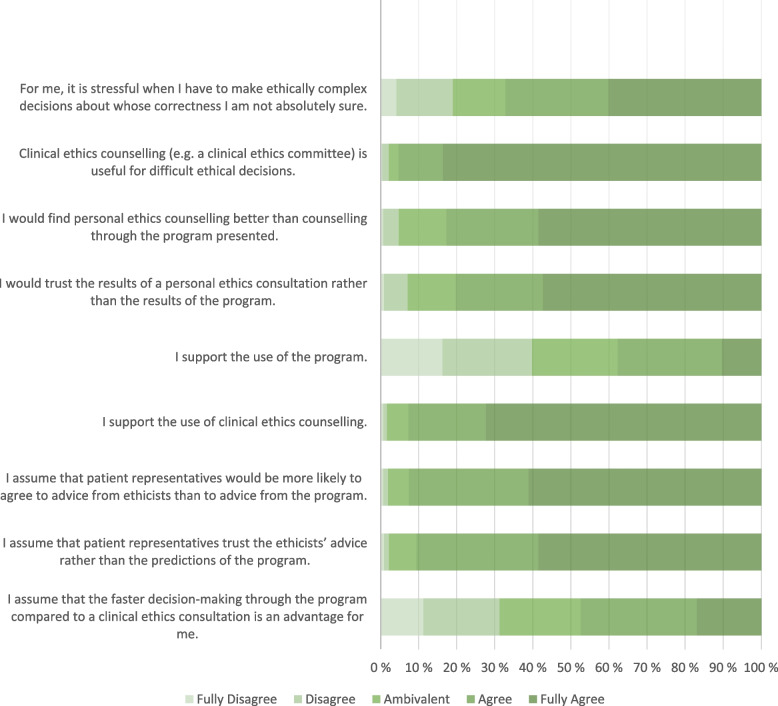
Responses to statements regarding clinical ethics support services and support through artificial intelligence

In contrast to the generally sceptical view on AI-driven preference prediction, the participants had a rather positive opinion on CESS; the answers to each individual item are depicted in Fig. 2.

The CESS were found to be useful in ethically difficult situations by the majority of physicians. The more a participant perceived ethically difficult situations as stressful, the more they agreed to the usefulness of CESS (ρ = 0.10, *p* = .033). This was especially true for women, who found the said situations more stressful (AM ± SD: 4.09 ± 1.12, *n* = 122) than men (3.73 ± 1.227, *n* = 276). Furthermore, the age had a significant impact on the perceived need for human ethics support, as participants tended to prefer CESS to the use of PPP more strongly the older they were (ρ = 0.10, *p* = .035). The more a participant was in favor of CESS, the more they believed patient representatives to think the same way (ρ = 0.34, *p* < .001).

### Coverage of CESS

Just over four-fifths (81.8%) of the participants reported that their respective clinics offered ethical support in the form of ethics counselling to healthcare providers and patient representatives. Out of those that have access, 59.3% had already made use of CESS, whereas 91.8% of those without access saw a need for establishing CESS. Among the participants without any access to ethics support, those who wished for the option were more likely to find ethically complex situations stressful (4.27 ± 0.969; *n* = 66) than those who did not see a need for CESS (2.17 ± 1.472; *n* = 6). Only 6 respondents (or 1.5%) out of the sample of 400 physicians have neither access nor the desire for ethics support in their clinical routine.

### Opportunities and risks for artificial intelligence in patient preference prediction

The participants were asked in the questionnaire’s free-text section to elaborate on the chances and risks they anticipate in the use of AI-driven patient preference prediction. The great majority of respondents provided substantial answers to at least one of the free-text questions (*n* = 394), ranging from single aspects to in-depth written responses. As a general result, 21 physicians saw no new opportunities to be established through AI, whereas only 3 participants saw that it introduced no risks.

The chances most frequently discussed were the increased speed of decision-making or aid thereof (*n* = 122), ensured objectivity and decreased bias in the decision-making process (*n* = 88), and general procedural benefits (*n* = 50). Perceived risks emerged mainly in relation to the de-humanization of decision-making processes (*n* = 146), the overreliance of (inexperienced) physicians on the proposed tools (*n* = 55) and incorrect predictions provided by the AI (*n* = 46). Examples of the most common reasons given can be found in Table 3.

**Table 3:** Pro and contra reasons for using AI-driven patient preference prediction

Pro	Contra
***Increased Speed***	***De-Humanization***
* ■ “Time saving with quick evaluation of a situation”*	* ■ “A decision based on demographic data is precisely not an individual one”*
* ■ “Faster decision-making, e.g. in triage situations”*	* ■ “Too much generalization – too little attention paid to individual patient needs”*
* ■ “Rapid availability in borderline situations without the possibility to wait for ethics consultation or discussion among colleagues”*	* ■ “Statistical programs run the risk of only depicting the peak of the Gaussian distribution”*
***Ensured Objectivity***	***Overreliance***
* ■ “Possibility of an objective collection of the data”*	* ■ “Blind and unreflected trust in the program”*
* ■ “Assessment outside my medical goals and philosophy of life/own quality of life”*	* ■ “The decisions of a computer cause a false sense of security for the decision-makers and can secondarily lead to conflicts of conscience”*
* ■ “Independence from ethical currents and schools or religious norms”*	* ■ “What I see above all is the risk that the program will be presumed to be responsible and one ceases to think for themselves and to deal with the patient and his or her individual situation”*
***Procedural Benefits***	***Incorrect Predictions***
* ■ “Suggestions from the AI can support the decision-making process”*	* ■ “Decisions that are too complex for an AI can lead to incorrect advice”*
* ■ “AI could, through automation and standardization, identify and present aspects that would otherwise not be considered”*	* ■ “Potentially worse/less accurate decision than by the doctor”*
* ■ “Additional component and further perspective to be able to put the decision on a broad basis”*	* ■ “A machine could come to wrong conclusions in borderline cases”*

Some participants provided opinions such as “Irrational decisions are sometimes also legitimate, but are not considered here.” They highlight that patients have a right to medical irrationality, which could, however, undermine one of the main advantages of AI: the objectivity of its suggestions. By being objective per se, the program may override the right to irrationality and enforce the feared loss of individuality in an entirely individualized field such as clinical ethics.

Furthermore, one participant brought up the fact that despite its proposed objectivity, AI may lead to “discrimination against people with disabilities,” as “many healthy people cannot imagine living like this, while people with disabilities often report a good quality of life and would very much like to have maximum therapy.”

Other participants pointed to a loss of the main advantages of ethical case review when automated procedures were employed: “Namely, that one pauses as a treatment team and relatives, steps out of the factual, temporal, procedural, personnel and, above all, monetary constraints and intensively consider the question of whether what one is doing here is good, is meaningful, and is even desired by the person concerned.” Doctors are aware that clinical ethics counselling offers more than just “yes or no” answers.

## Discussion

### Principal results

The participants showed hesitance towards the use of an AI-driven PPP, fostered by the suspicion that it may lead to de-humanisation in the ethical deliberational process. The lack of explicability of these systems and the anticipated inability of AI to mimic the process of ethical deliberations were weighed against advantages, such as time-savings in ethical support and the objectivity of decisions. The CESS enjoy a higher reputation than digital support systems, and are available in a large majority of hospitals.

### AI-driven decision support in healthcare

Clinical Decision Support Systems (CDSS) have many similarities to the proposed PPP, as they share a similar purpose in assisting physicians and other stakeholders in clinical decisions with implications for patients’ health and well-being. It is, therefore, reasonable to compare the broad debate on CDSS with our findings on patient preference prediction. Lambert et al. assess the acceptance of AI, predominantly integrated in CDSS, amongst healthcare providers in an integrative review. Reoccurring topics, such as the hope for increased accuracy of decisions, time-savings and improved efficiency are brought up in both discussions. Additionally, the issue of transparency and explicability is discussed in relation to CDSS. The issue of unclear responsibility and liability for recommendations made by AI was rarely raised by the study participants, but thoroughly represented in the discussion of CDSS [36].

The European Union Ethics Guidelines for Trustworthy Artificial Intelligence propose a framework of seven key requirements which developers can adhere to in order to create AI models in line with ethical demands [37]. While the proposed requirements line up remarkably well with the hopes and risks discussed by the participants of our survey, the participants rarely addressed the requirement of human agency and oversight. This requirement aims to clarify the relationship between man and machine, as it reads, “AI systems should empower human beings, allowing them to make informed decisions” [37]. If AI were tailored within the boundaries of clarified roles, this would allow the systems to be reliably seen as tools that help to enhance professionals’ abilities, rather than as a rival trying to render the user redundant. The aforementioned human oversight goes hand in hand with the prerequisite of transparency, which not only refers to the often mentioned explicability of the algorithms per se, but also the transparency that AI is being used at all, which is important to disclose to patients to maintain a trustful relationship [38].

### Autonomy

The participants’ wide-ranging attitudes show that AI should not be categorically rejected for preference prediction. The broad spectrum may be related to normatively important aspects that different participants assess differently, such as individual conceptions of autonomy. On the one hand, preference prediction through AI seems futile for physicians who conceive autonomy as sovereignty [39]. This is because the program does nothing to support the patient’s capabilities to be informed, to consider the advantages and risks of different ways of action, as well as to form a personal decision and express it. From this perspective, however, surrogate decision-making should be seen as equivalently aimless, because although the agents change, the outcome stays the same. On the other hand, AI may actually help to improve the quality of predictions and, thereby, patient autonomy for physicians who conceive autonomy as authenticity [39], i.e. that decisions are coherent with the preferences of patients. Limitations, however, arise for patients whose preferences are “atypical” for their age, gender, etc., or who are not well represented in the training material, for example, for migration or cultural reasons.

The survey participants mentioned various potential infringements that the use of AI could impose on patient autonomy, such as incorrect predictions or its utilization despite low acceptance amongst stakeholders. In their related article, Jardas et al. succeed in debunking a multitude of these worries [40]. They state, for example, that in contrast to the claims that patient preference prediction suffers from low acceptance among its users, a sizeable percentage of patients surveyed preferred the use of a PPP to surrogate decision-making, or, at least, wished for their surrogates to benefit from the support of one [41]. Furthermore, they refuted the allegations that the statistical nature of the preference prediction violates patient autonomy in general. The violation would only take place in the “hard default” case when the PPP is used as “naked” statistical evidence. Jardas et al. invalidate an important fear in highlighting that additional consultations of physicians and surrogates usually enrich the decision-making process.

### Explicability

An important reason for the scepticism towards AI appears to be the missing explicability of the results. Explicability is a highly debated topic regarding CDSS in general [42]. More than 70% of our survey participants fully agreed that they want to be able to understand and retrace the steps the algorithm has taken to reach its conclusion. The black-box character of some AI models complicates this requirement. Professional bodies, such as radiological associations, justify the explicability mostly as a vehicle for the principle of non-maleficence because there is a need to reduce harms inflicted by performance errors of medical AI [43]. General solutions for explainable AI in medicine are inherently interpretable models, feature visualization, prototypes, counterfactuals or feature attribution [38].

### Opinions of different stakeholders

So far, there have been only initial insights into the opinions of patients and patient representatives towards AI-driven patient preference prediction: Wendler et al. report that patients support the use of a preference predictor, given that it helps to improve the accuracy of predictions and lowers the burden imposed on their decisional representatives [41]. Our findings show that the physicians surveyed believed in the usefulness of a preference predictor for mitigating surrogate stress, whereas the assessment towards the accuracy of predictions and its impact on actual patient autonomy was mixed. Furthermore, the allocation of roles in the human-machine interaction was of importance to the patients questioned, as they preferred the prediction made by their human surrogates to the AI-prediction in cases in which the two parties had differing opinions [41]. This further backs up the assumption that, analogously, physicians care about their role in decision-making, and that this point of critique needs further clarification.

### Limitations

We acknowledge two main limitations of our work. Firstly, the form of recruitment might have influenced the sample. The physicians reached were presumably mostly tech-savvy because advertising and registration was exclusively online. While this may be an advantage for our study, as knowledge about aspects of digital issues allows for more nuanced opinions on the technology at hand, it also has the potential to distort the findings in favor of any digital innovation if the savviness is drowned out by enthusiasm. Additionally, online-based recruitment may discriminate against certain demographic groups, as can be seen in the age distribution of the sample.

Secondly, the uneven distribution of gender in our sample may pose a further limitation. Although no significantly varying appraisals of different aspects related the research question between the different genders were found, a more representative gender distribution might have led to opinions reported differently and, therefore, more representative findings.

Thirdly, the lack of randomisation of the order of items within the questionnaire may lead to biases in the answers. Randomisation was deliberately omitted in order to keep the structure of the topic blocks within the questions and general comprehensibility. The order, however, may have influenced the respondents’ answering behavior.

## Conclusions

German physicians frequently in contact with incapacitated patients were hesitant regarding the use of AI to predict patient preferences, while demonstrating a high esteem for CESS. The driving factors behind their reluctance are the fear of compromised individuality and humanity, the limited explicability in the decision-making procedure and the proposed inability of AI to encompass the full process of ethical deliberations. Even though advantages, such as time-savings in ethical support and the objectivity of decisions, are conceded to the novel technology, the participants expect patients and their representatives to share their scepticism toward automated preference prediction and the appraisal of CESS.

If preference prediction through AI is desired in the clinical routine, further effort should be invested in the progression of explicable AI. The traceability of the inner workings of the algorithms may enable the users affected to put their trust in the procedure and appreciate the outcome. Furthermore, additional clarification and regulation of the envisaged roles of physician and AI in their mutual endeavor of improved patient care conceivably mitigates the reservations physicians hold towards their digital implementation, as they fear for their own significance and responsibility in the decision-making process.

Although physicians are important stakeholders in dealing with incapacitated patients, what matters even more are the patients themselves, their relatives and other people acting as surrogates. Thorough research into the opinions, wishes and fears of those mainly affected is needed, especially against the background of the recent progress of AI development, to ensure apt development and approval of AI in their clinical experience. Additional enquiry into the conditions for the AI’s predictions to be seen as reflecting actual individuality and the steps required for stakeholders to trust AI is necessary before it can be used in everyday clinical practice.

### Supplementary Information

Supplementary Material 1.

## Data Availability

The datasets used and analysed during the current study are available from the corresponding author on reasonable request.

## Abbreviations

AI: Artificial intelligence
CESS: Clinical ethics support services
CDSS: Clinical decision support systems
LLM: Large language model
METHAD: Medical ethical advisor
PPP: Patient preference predictor
