# Supplementary material for: Artificial Intelligence to support ethical decision-making for incapacitated patients: a survey among German anesthesiologists and internists
Source: BMC Med Ethics. 2024 Jul 18;25:78. doi: 10.1186/s12910-024-01079-z (PMC11256615; doi:10.1186/s12910-024-01079-z)
Supplement: Supplementary file 1 — Supplementary Material 1. [file 12910_2024_1079_MOESM1_ESM.docx]

Questionnaire

**Questionnaire on AI-based decision support for non-consenting patients**

Commissioned by: Hannover Medical School (MHH)

Field start: February 2023

Sample: n=400 clinically active specialists, of which n=200 internists and n=200 anesthetists

Study objective: To investigate the attitude of medical specialists towards AI-based decision support for patients who are not capable of giving consent.

**SCREENING**

**S1. Subject area**

In which specialty do you work?

1) Anesthesiology

2) General medicine

3) Ophthalmology

4) Radiology

5) Gynecology

6) Internal medicine

7) Other, namely ________

**S2. Place of work**

Where do you currently work primarily?

1) Individual practice

2) Joint practice / group practice

3) Medical care center

4) Clinic

5) University hospital

6) Rehabilitation clinic

7) Authorities / Corporations

8) Other

**MAIN SURVEY**

**F0. Introduction**

The following text provides a brief introduction to the topic of the survey. Please read the paragraph carefully. You will then be asked the questions relevant to the survey.

Imagine: In a few years, your hospital information system will contain a program to help you when making complicated ethical decisions (e.g. limiting therapy in intensive care). These are decisions for patients who cannot express their will themselves.

Based on clinical, demographic and personal characteristics from the medical record, the program predicts the preferences of incapacitated patients for an upcoming therapy decision. The prediction of the program is based on empirically collected training data processed by artificial intelligence.*

The predictions generated by the program can be utilized to assist doctors or patient representatives in making decisions when there is no valid advance directive and the patients’ preferences are largely unknown.

*Artificial intelligence (AI) is the ability of a machine to imitate human competencies, such as logical thinking, learning, planning and creativity. It enables technical systems to find correlations in the data provided without explicit guidance and, based on these, to independently optimize their learning and work processes as well as their results.

**F1. Statements 1**

To what extent do you agree with the following statements about the program just described?

1. The increased accuracy of predictions provided by the program helps to preserve patient autonomy.

2. Individual patient preferences can be derived from statistical correlations on which the program's predictions are based.

3. The program ensures that patients receive the treatment they would have wished for in their situation.

4. In order to preserve a patient's autonomy, the decision may not necessarily be the right one, but must have been made for the right reasons.

5. The information provided by the program offers a good basis for decision-making.

**F2. Statements 2**

To what extent do you agree with the following statements about the program just described?

1. The program is able to reduce the moral burdens of patient representatives.

2. The program is able to reduce the moral burdens of doctors.

3. Artificial intelligence can fully encompass the ethical decision-making process.

4. The program reinforces social injustices that result from the underlying data.

5. In order to trust the program's predictions, I must be able to understand how it arrived at them.

**F3. Ethics counselling**

Does your hospital offer the possibility for doctors or patient representatives to receive ethics counselling?

1) Yes

2) No

**F3.1 Ethics counselling available**

Have you already made use of ethics counselling in your hospital?

1) Yes

2) No

**F3.2 Ethics counselling not available**

Would you like your hospital to offer ethics counselling for doctors and patient representatives?

1) Yes

2) No

**F4. Statements 3**

The following section is about your personal attitude towards ethics and ethics counselling in medicine. To what extent do you agree with the following statements about ethics and ethics consultation?

1. For me, it stressful when I have to make ethically complex decisions about whose correctness I am not absolutely certain.

2. Clinical ethics counselling (e.g. a clinical ethics committee) is useful for difficult ethical decisions.

3. I would find personal ethics counselling better than counselling through the program presented.

4. I would trust the results of a personal ethics consultation more than the results of the program.

**F5.Statements 4**

To what extent do you agree with the following statements about ethics and ethics counselling?

1. I support the use of the program.

2. I support the use of clinical ethics consultation.

3. I assume that patient representatives would be more likely to agree to advice from ethicists than to advice from the program.

4. I assume that patient representatives trust the ethicists’ advice more than the predictions of the program.

5. I assume that the faster decision-making through the program compared to clinical ethics consultation is an advantage for me.

**F6. Opportunities**

What opportunities and risks do you see in the use of artificial intelligence for predicting patient preferences?

Please describe as precisely as possible which opportunities and risks you see.

**SOCIODEMOGRAPHICS**

**So1. Gender**

Are you...?

1. Female

2. Male

3. Non-binary

**So2. Age**

How old are you?

___ Years

**So3. State**

Please indicate in which federal state you practice.

1) Baden-Wuerttemberg

2) Bavaria

3) Berlin

4) Brandenburg

5) Bremen

6) Hamburg

7) Hesse

8) Mecklenburg-Western Pomerania

9) Lower Saxony

10) North Rhine-Westphalia

11) Rhineland-Palatinate

12) Saarland

13) Saxony

14) Saxony-Anhalt

15) Schleswig-Holstein

16) Thuringia
